# AI Efficiency in Dentistry: Comparing Artificial Intelligence Systems with Human Practitioners in Assessing Several Periodontal Parameters

**DOI:** 10.3390/medicina61040572

**Published:** 2025-03-23

**Authors:** Oana-Maria Butnaru, Monica Tatarciuc, Ionut Luchian, Teona Tudorici, Carina Balcos, Dana Gabriela Budala, Ana Sirghe, Dragos Ioan Virvescu, Danisia Haba

**Affiliations:** 1 Department of Biophysics, Faculty of Dental Medicine, “Grigore T. Popa” University of Medicine and Phamacy, 700115 Iasi, Romania; 2 Department of Prosthodontics, Faculty of Dental Medicine, “Grigore T. Popa” University of Medicine and Pharmacy, 700115 Iasi, Romania; 3 Department of Periodontology, Faculty of Dental Medicine, “Grigore T. Popa” University of Medicine and Pharmacy, 700115 Iasi, Romania; 4 Department of Oral Health, Faculty of Dental Medicine, “Grigore T. Popa” University of Medicine and Pharmacy, 700115 Iasi, Romania; 5 Department of Pediatric Dentistry, Faculty of Dental Medicine, “Grigore T. Popa” University of Medicine and Pharmacy, 700115 Iasi, Romania; 6 Department of Dental Materials, Faculty of Dental Medicine, “Grigore T. Popa” University of Medicine and Pharmacy, 700115 Iasi, Romania; 7 Department of Dental Radiology, Faculty of Dental Medicine, “Grigore T. Popa” University of Medicine and Pharmacy, 700115 Iasi, Romania

**Keywords:** periodontal status, alveolar bone resorption, bone loss, periodontal pocket, AI, dental imaging, periodontitis

## Abstract

Artificial intelligence (AI) is increasingly used in healthcare, including dental and periodontal diagnostics, due to its ability to analyze complex datasets with speed and precision. *Backgrounds and Objectives:* This study aimed to evaluate the reliability of AI-assisted dental–periodontal diagnoses compared to diagnoses made by senior specialists, specialists, and general dentists. *Material and Methods:* A comparative study was conducted involving 60 practitioners divided into three groups—general dentists, specialists, and senior specialists—along with an AI diagnostic system (Planmeca Romexis 6.4.7.software). Participants evaluated six high-quality panoramic radiographic images representing various dental and periodontal conditions. Diagnoses were compared against a reference “gold standard” validated by a dental imaging expert and senior clinician. A statistical analysis was performed using SPSS 26.0, applying chi-square tests, ANOVA, and Bonferroni correction to ensure robust results. *Results:* AI’s consistency in identifying subtle conditions was comparable to that of senior specialists, while general dentists showed greater variability in their evaluations. The key findings revealed that AI and senior specialists consistently demonstrated the highest performance in detecting attachment loss and alveolar bone loss, with AI achieving a mean score of 6.12 in identifying teeth with attachment loss, compared to 5.43 for senior specialists, 4.58 for specialists, and 3.65 for general dentists. The ANOVA highlighted statistically significant differences between groups, particularly in the detection of attachment loss on the maxillary arch (F = 3.820, *p* = 0.014). Additionally, AI showed high consistency in detecting alveolar bone loss, with comparable performance to senior specialists. *Conclusions:* AI systems exhibit significant potential as reliable tools for dental and periodontal assessment, complementing the expertise of human practitioners. However, further validation in clinical settings is necessary to address limitations such as algorithmic bias and atypical cases. AI integration in dentistry can enhance diagnostic precision and patient outcomes while reducing variability in clinical assessments.

## 1. Introduction

By providing new resources for helping doctors in diagnosis and treatment, artificial intelligence (AI) is revolutionizing the healthcare industry [1]. These developments have not escaped the dentistry field, especially in the domains of periodontal and dental diagnostics [2,3]. Effective patient care relies on accurate diagnoses, consistent results, and little wasted time; AI holds great potential in this area [4]. However, serious doubts regarding AI’s reliability in contrast to human knowledge have been raised by its integration into clinical practice [5].

In recent years, AI has demonstrated significant potential in various branches of dentistry, from caries detection to orthodontic treatment planning and periodontal disease management [6]. AI algorithms, particularly those based on deep learning and neural networks, are adept at analyzing large datasets, including radiographs, intraoral scans, and histological images [7,8]. In periodontal care, these systems can identify patterns and markers indicative of conditions such as gingivitis, periodontitis, and bone loss, often with remarkable speed and accuracy [9]. Algorithms that can quickly analyze and understand complicated clinical data, such as radiographic pictures and patient records, have emerged as a consequence of the fast development of AI technology [10,11,12]. When it comes to managing chronic illnesses, this expertise is invaluable. Precise monitoring and early identification are of the utmost importance [13].

Beyond periodontal diagnosis, AI has been successfully applied in multiple fields of dentistry, significantly contributing to improved patient outcomes and clinical efficiency [14]. In restorative and prosthetic dentistry, AI is used to assist in the design of dental prostheses, ensuring better adaptation and function [15]. In endodontics, AI-based systems help in detecting periapical lesions, classifying root canal morphology, and predicting treatment success [16]. In orthodontics, AI plays a crucial role in cephalometric analysis and treatment simulation, providing personalized treatment plans for patients [17]. Furthermore, in oral and maxillofacial surgery, AI aids in diagnosing cysts, tumors, and fractures, facilitating surgical planning and precision [18].

Another important contribution of AI in dentistry is its role in predictive analytics and personalized treatment [19]. Machine learning models can analyze a patient’s medical history, lifestyle factors, and genetic predisposition to estimate the risk of developing dental diseases, allowing for early intervention and tailored prevention strategies [20]. AI is also used in the automation of administrative tasks, optimizing appointment scheduling, patient record management, and workflow efficiency in dental clinics [21].

Through its diverse applications, AI continues to enhance the accuracy, efficiency, and accessibility of dental care. While its integration into daily clinical practice is still evolving, the growing body of research demonstrates that AI has the potential to revolutionize modern dentistry by reducing diagnostic errors, improving treatment planning, and increasing overall patient satisfaction [22].

Despite these developments, implementing AI in dentistry presents obstacles to overcome [23]. Significant challenges continue to exist, including algorithmic bias, poor generalizing across different patient groups, and the necessity for high-quality, labeled training data [24]. Furthermore, while AI systems excel in pattern recognition, they may struggle with unique cases or complicated clinical circumstances that need nuanced judgment [25]. These limitations highlight the need to compare AI technologies to the knowledge of human practitioners. Whether these technologies can match or even outperform the diagnostic skills of human dentists remains an open issue [26,27].

Dental knowledge is developed through formal education, on-the-job training, and patient care. Many people believe that the most accurate diagnoses are made by senior doctors due to their wealth of experience and excellent judgment. While specialists are great in their own fields, general dentists are great at providing the more holistic treatment that patients need. The performance of AI systems has to be measured against these varied levels of human competence [28,29,30].

This study builds upon prior research by providing a comparative analysis of AI-assisted periodontal diagnostics in relation to human practitioners at different expertise levels. While previous studies have primarily focused on AI’s technical capabilities, such as image processing and segmentation, our research extends the evaluation to real-world clinical applications by assessing AI’s diagnostic accuracy alongside general dentists, specialists, and senior practitioners [31,32].

By systematically comparing AI performance with varying levels of human expertise, we aim to bridge the gap between AI validation in controlled settings and its practical implementation in clinical dentistry. Unlike prior studies that often rely on retrospective image datasets, this research incorporates a structured diagnostic protocol, ensuring a more realistic clinical assessment [33,34]. Furthermore, by utilizing Planmeca Romexis, an AI-powered diagnostic platform with deep learning capabilities, our study not only evaluates AI’s effectiveness but also examines its potential role in reducing diagnostic variability among practitioners.

These findings contribute to the ongoing discussion on AI integration in dentistry, offering evidence-based insights into its strengths and limitations in periodontal diagnostics.

The increasing amount of data indicating that AI may surpass traditional diagnostic methods in many dental applications is the driving force behind this study. When it comes to dental problems, treatment planning, and result prediction, AI algorithms, especially those using deep learning frameworks and convolutional neural networks, have proven to be more accurate and efficient [35,36]. Despite these encouraging developments, there are still many obstacles to overcome before AI can be fully integrated into everyday dentistry practice. These include limits in methodology, restrictions imposed by regulations, and ethical concerns [37].

To determine the presence and amount of bone loss, radiography is often used as a noninvasive supplement to clinical examination during comprehensive periodontal assessment [38,39,40]. Clinical attachment levels and radiographic bone loss are utilized together. Artificial intelligence models for identifying alveolar bone loss from radiographs have an accuracy ranging from 73.4% to 99%, according to a recent systematic study [41].

The evaluation of periodontal status is a fundamental aspect of diagnosing and managing periodontal disease. Its key clinical indicators include pocket depths, clinical attachment levels, and radiographic bone levels. Accurate evaluation is essential for identifying the stage and severity of periodontal disease, guiding treatment planning, and monitoring disease progression over time. Artificial intelligence technologies automate the evaluation of radiographs and clinical data, which is a crucial part of this procedure. Deep learning algorithms allow AI to spot periodontal disease symptoms, including radiographic abnormalities and early bone loss, which may be overlooked in manual evaluations.

By comparing the diagnostic outputs of AI systems with those of human practitioners across varying levels of expertise, this study sought to identify both the strengths and limitations of AI in dental and periodontal care. The methodology involved a detailed assessment of diagnostic outcomes using clinical cases, ensuring a robust and practical evaluation framework. This study adds to the existing literature on artificial intelligence in healthcare by providing a thorough comparison, which, in turn, can help doctors, policymakers, and legislators.

Ultimately, we aimed to determine whether AI can serve as a complementary tool to human expertise or if its limitations necessitate caution in its clinical application. This analysis is crucial for shaping the future of AI integration in dentistry and ensuring that technological advancements align with the highest standards of patient care.

## 2. Materials and Methods

Research on the use of AI approaches in periodontics has been growing at an exponential rate; therefore, the purpose of this comparative analytical study was to evaluate the accuracy of AI-assisted dental–periodontal imaging versus assessments made by general dentists, specialists, and senior specialists. The statistics were derived from the clinical records of patients who received treatment at the M Kogalniceanu Iasi Educational Centre between 2022 and 2023. This study received approval from the Institutional Review Board of UMF Gr.T. Popa, Iasi (No. 23/8.03.2023). All participants provided informed consent before taking part in the study, and the data used was anonymized to protect patient confidentiality. This study was approved by the Ethics Committee of the Faculty of Dental Medicine Gr.T. Popa Iasi.

This study evaluates the performance of AI in assessing periodontal parameters using radiographic images, specifically focusing on bone loss and pocket depth, rather than a comprehensive periodontal diagnosis, which requires both clinical and radiographic evaluation.

The research involved a group of 60 dentists chosen based on their professional skills. The groups consisted of the following:

✓ 20 general dentists.
✓ 20 specialists in periodontology.
✓ 20 senior specialists.
✓ The AI system used for diagnosis (Planmeca Romexis,6.4.7.version Helsinki, Finland).

The Planmeca Romexis^®^ 6.4.7.version software, Helsinki, Finland platform provides a complete solution for dental imaging, diagnostics, and treatment planning. It supports a wide range of imaging modalities, including 2D, 3D, and CAD/CAM, making it appropriate for clinics of various sizes and specialties. The program was recently updated with new AI-powered capabilities for 2D and 3D imaging. These technologies use artificial intelligence to create recommendations, but the final decision-making power always stays with the clinician. Romexis^®^ Smart is an optional feature of the Romexis 3D imaging module.

It automatically segments and recognizes anatomical structures such as the skull, soft tissues, teeth, nerves, jaws, airways, and sinuses. The new functionality makes the program much easier and faster to use, and the visualization of the anatomical structures makes it an ideal tool for communicating with patients. Automated 2D image analysis for panoramic and intraoral X-Rays provides support that can help dentists make better diagnoses, enhance patient communication, and improve oral health outcomes. AI analysis helps identify signs of various dental pathologies and other treatable conditions found in dental radiographs, including hard-to-detect issues. The program can detect, among others, the following:

▪ Potential cavities, recommending the best treatment option, such as a filling, inlay, onlay, crown, endodontic therapy, or extraction. The findings are emphasized with color and language, creating a readily comprehensible impartial view that contributes to patient trust. This enables immediate treatment and long-term health benefits through improved patient retention.
▪ The proportion or presence of bone resorption.
▪ Furcation lesions.
▪ Periodontal pockets.
▪ Symptoms of different dental diseases and other treatable conditions, including difficult-to-detect issues like the first symptoms of periapical radiolucency.

### 2.1. Study Stages

#### 2.1.1. Case Selection

Six high-quality radiological images (OPGs) were selected, representing various periodontal conditions (e.g., bone loss, periodontal pockets, and furcation injuries).

OPGs allow for the detection of other conditions, such as sinus involvement or jaw abnormalities, which could influence periodontal health but might not be captured in a localized periapical image. In the context of this study, the selection of high-quality OPGs pre-validated by dental imaging experts ensured reliable diagnostic benchmarks.

The parameters evaluated were selected to reflect periodontal health using panoramic radiographic images (OPGs). Alveolar bone loss was measured by determining the height of the alveolar bone relative to the cemento–enamel junction (CEJ). The assessment included horizontal bone loss (uniform reduction of bone height along the dental arch) and vertical bone defects (angular defects associated with periodontal pockets or furcation lesions). The severity was classified as mild (<3 mm from the CEJ), moderate (3–5 mm), or severe (>5 mm).

Clinical attachment loss was indirectly assessed by observing the destruction of supporting tissues visible on radiographs. This included identifying the number of teeth affected by generalized and localized attachment loss based on the extent of tissue destruction visible on the radiographs, with corresponding severity grades.

Additionally, radiographic indicators of periodontal pockets, such as angular bone defects, radiolucent gaps, and irregularities in the alveolar crest, were evaluated.

**Figure 1:**
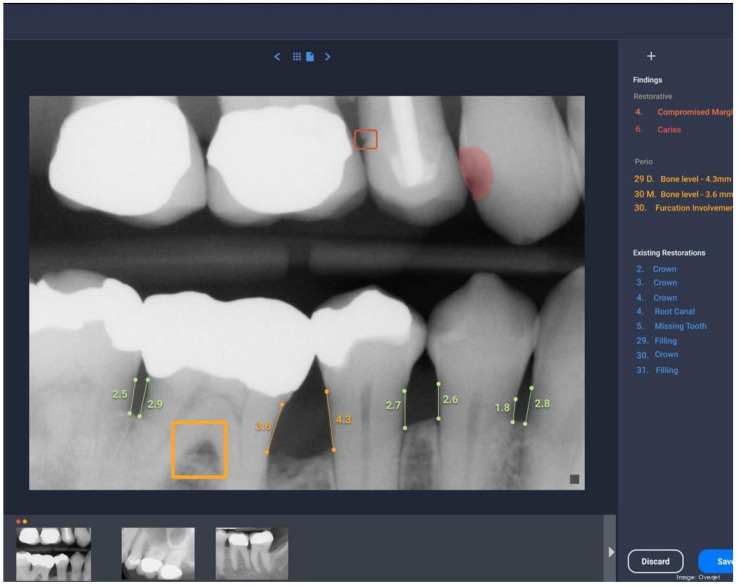
Measurements using the software Romexis^®^ Smart and AI module.

**Figure 2:**
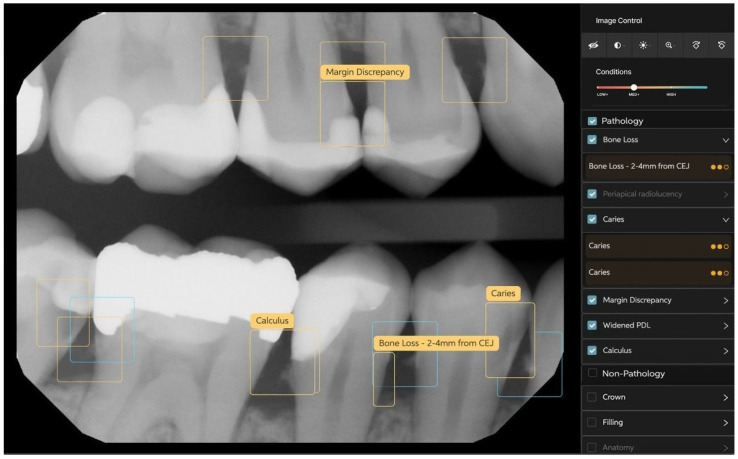
Identification of several periodontal aspects using Romexis^®^ Smart and AI.

Interproximal bone levels were measured by calculating the linear distance between the CEJ and the alveolar crest in interproximal spaces, with an analysis of symmetry between adjacent teeth to identify localized patterns of bone loss. These parameters provide a robust framework for assessing AI performance and its clinical relevance in periodontal diagnostics, as can be seen in Figure 1 and Figure 2 below.

The cases were pre-validated by a dental imaging expert and a senior dentist to ensure a reliable reference.

#### 2.1.2. Participant Intervention

Each participant individually evaluated the set of images without access to the patient’s clinical history and formulated a diagnosis for each image.

The AI-generated assessment in this study was produced using a deep learning algorithm embedded within the Planmeca Romexis^®^ platform. This algorithm was specifically trained on datasets comprising panoramic radiographs validated by experienced dental imaging experts. Deep learning, as a subset of machine learning, utilizes convolutional neural networks (CNNs) to process image data, enabling the algorithm to identify patterns indicative of periodontal conditions such as alveolar bone loss, periodontal pockets, and furcation lesions.

The training data included labeled examples that reflected a diverse range of dental conditions to improve the model’s ability to generalize across various clinical scenarios. The training and validation processes were structured to minimize overfitting, ensuring the model’s robustness when applied to unseen clinical cases. The AI model’s reliability was validated through a two-step approach: (1) training and validation using a diverse dataset of panoramic radiographs, and (2) comparative assessment against expert evaluations. The AI system utilized in this study (Planmeca Romexis) was trained on a large dataset of labeled dental radiographs, validated by experienced dental imaging specialists to ensure accurate ground truth labels.

To assess AI robustness, the system underwent internal cross-validation, wherein the dataset was randomly split into training (80%) and validation (20%) subsets to optimize model performance. Additionally, to determine its generalizability, the AI’s diagnostic outputs were compared against an external test set of clinical cases and evaluated by a panel of senior specialists. The final AI predictions were then analyzed for agreement with expert diagnoses, using statistical validation techniques to assess performance metrics such as accuracy, sensitivity, and specificity.

Furthermore, the system employs advanced preprocessing techniques to enhance image clarity and highlight diagnostic features, such as radiographic bone loss, that might be overlooked during manual evaluation.

#### 2.1.3. Performance Evaluation

Assessments from each group of practitioners and the AI system were compared against the reference assessment (“gold standard”).

To ensure the consistency of periodontal evaluations among human examiners, we assessed inter-examiner reliability using the Intraclass Correlation Coefficient (ICC) and Cohen’s kappa statistic. The ICC was used for continuous variables, such as the measurement of alveolar bone loss and attachment loss, while Cohen’s kappa was applied to categorical assessments, such as the presence or absence of periodontal pockets and furcation lesions.

The ICC values indicated strong agreement among senior specialists (ICC = 0.91), moderate-to-strong agreement among specialists (ICC = 0.84), and moderate agreement among general dentists (ICC = 0.72). Cohen’s kappa results demonstrated substantial agreement among senior specialists (κ = 0.87), while specialists and general dentists showed moderate agreement (κ = 0.79 and κ = 0.68, respectively). These findings confirm high diagnostic consistency among experienced practitioners while highlighting some variability among less-experienced evaluators.

#### 2.1.4. Statistical Analysis

The collected data were statistically analyzed using SPSS 26.0 software (IBM, Armonk, NJ, USA). The results are presented as frequencies and mean values. To demonstrate significant differences between groups regarding periodontal evaluation, statistical tests such as the chi-square test and ANOVA were applied.

Due to the small sample size, the Bonferroni test was employed to adjust for multiple comparisons, reducing the risk of false-positive results (Type I error). The Bonferroni method ensured that the identified differences between groups were robust and not random artifacts caused by numerous tests.

The significance level was set at *p* < 0.05 to validate statistical reliability.

## 3. Results

### 3.1. Assessing Clinical Attachment

The analysis of the results revealed clear differences between the groups in identifying cases of attachment loss. AI and senior practitioners consistently had the highest averages, indicating an increased ability to detect these pathologies.

The average for teeth with attachment loss was 6.12 for AI, 5.43 for senior practitioners, 4.58 for specialists, and 3.65 for general dentists. On average, AI and senior practitioners provided 5.85 and 5.33 points to the maxillary arch, respectively, whereas specialists and general dentists contributed 4.13 and 3.71 points less, respectively. In a comparable manner, regarding the mandible, AI had the best average (5.72), followed by specialists (5.04) and senior practitioners (5.62), while general dentists remained the least involved, with 4.15. The results indicate that AI and senior practitioners are the most skilled at detecting cases of attachment loss. In contrast, specialists and general dentists appear to be less involved, maybe because they focus on fewer difficult patients.

The ANOVA analysis indicated significant differences between groups in identifying teeth with attachment loss and attachment loss on the maxillary arch but not for attachment loss on the mandibular arch. For teeth with attachment loss (F = 3.283, *p* = 0.027), AI and senior practitioners, who had the highest averages according to the previous charts, were more involved in identifying complex cases. Similarly, for attachment loss on the maxillary arch (F = 3.820, *p* = 0.014), AI (5.85) and senior practitioners (5.33) dominated, while specialists and general dentists showed lower average values (as shown in Table 1).

For mandibular attachment loss, the differences between groups were not significant (F = 1.802, *p* = 0.156), indicating a more uniform performance across participant types, although AI and senior practitioners continued to have the highest averages, as can also be seen in Table 1. These results highlight that AI and senior practitioners are the most involved and efficient in identifying attachment loss overall, with greater expertise in complex cases, while differences for mandibular attachment loss are less pronounced and do not show significant variation among participants.

For teeth with attachment loss, multiple comparisons showed that the differences between groups were not statistically significant (*p* > 0.05), although there was a tendency for senior practitioners to identify more affected teeth than general dentists (mean difference = 1.77, *p* = 0.065) and specialists (mean difference = 0.84, *p* = 1.000). AI demonstrated performance comparable to that of senior practitioners, suggesting a relatively similar involvement in complex cases of attachment loss. For attachment loss on the maxillary arch, senior practitioners were significantly more efficient than general dentists (mean difference = 1.63, *p* = 0.047), while the differences compared to specialists and AI were not significant. AI showed better performance than general dentists (mean difference = 2.15, *p* = 0.098), but this difference was not statistically significant, as shown in Table 2.

Regarding attachment loss on the mandibular arch, the differences between groups were not significant (*p* > 0.05), but there was a tendency for senior practitioners to detect more cases than general dentists (mean difference = 1.47, *p* = 0.214), while AI demonstrated performance comparable to that of senior practitioners. These results in Table 2 highlight the essential role of senior practitioners and AI in identifying complex cases of attachment loss, particularly on the maxillary arch, whereas general dentists and specialists tended to be less involved in these cases. The trends observed confirm the differences in expertise and specialization among the types of participants.

### 3.2. Assessing Periodontal Pockets

Senior specialists demonstrated consistent and superior performance across all types of evaluations, exhibiting perfect uniformity in assessment. Specialists and AI showed comparable but inferior performance to senior specialists, with moderate variability across evaluations. General dentists, in contrast, recorded the weakest results, particularly for oblique periodontal pockets, where the average dropped to 0.3500. This highlights a clear need for additional support, either through training or the integration of AI-based technologies. Overall, AI showed promising potential for supporting groups with lower performance, as pointed out in Table 3 below.

The ANOVA test results show that for vertical and oblique periodontal pockets, there were significant differences between groups, highlighting the superiority of senior specialists in diagnosis. In contrast, at the level of general diagnosis of teeth with periodontal pockets, the variations between groups were not significant. These findings suggest that experience and expertise substantially contribute to identifying complex cases, such as vertical and oblique periodontal pockets. Moreover, AI could be valuable in reducing variability among lower-performing groups, such as dentists. All of these results are synthesized in Table 4 below.

The Bonferroni test results highlight significant differences between groups regarding the efficiency of detecting various types of periodontal space widening. When evaluating teeth with periodontal pockets, there were no statistically significant differences between groups (all *p*-values > 0.05). The differences between senior specialists and other participants were notable but not large enough to reach statistical significance, as shown in Table 5.

For vertical periodontal pockets, there was a significant difference between senior specialists and dentists (*p* = 0.001), with a mean difference of 0.50. This indicates a clear superiority of senior specialists in identifying this type of widening. Similarly, for oblique periodontal pockets, the difference between senior specialists and dentists was significant (*p* = 0.000), with a mean difference of 0.65, highlighting greater difficulty for dentists in this context. No significant differences were observed between AI and the other groups, suggesting that AI performance is comparable to that of specialists and dentists but inferior to that of senior specialists.

### 3.3. Evaluation of Periodontal Space Widening

Senior specialists proved to be the most efficient in detecting periodontal space widening, maintaining a consistent average of 1.00 with no variation across all analyzed cases. This highlights the high precision and reliability of this group. In contrast, specialists demonstrated moderately high efficiency, with an overall average of 0.90, but a slight decrease in performance in the maxilla (0.65), indicating a lower level of uniformity in evaluations. AI showed stable performance comparable to that of specialists, with an average of 0.8333 and moderate variability in the results, suggesting promising potential as a supportive diagnostic tool.

In contrast, dentists recorded the lowest performance, with an overall average of 0.65 and high variability between evaluations, particularly for the periodontal space in the mandible (0.50), as can be seen in Table 6. This highlights the need for improvement, either through additional training or by integrating technological tools to support diagnosis. Overall, the analysis revealed that experience plays a crucial role in detection efficiency, while also emphasizing that AI-based technologies can significantly contribute to reducing variability and improving diagnostic accuracy.

The ANOVA test indicated statistically significant differences between examiner groups in identifying periodontal space widening (widened PDL) at the general level, in the maxilla, and in the mandible. The results are illustrated in Table 7. For generalized periodontal space widening, an F-value of 7.756 and *p* = 0.000 indicate significant variations between groups, although the within-group variability was greater than the between-group variability. In the case of the maxilla, an F-value of 6.086 and *p* = 0.001 reveal moderate differences between examiners, suggesting slight variations in their approaches to identifying this pathology in the maxillary region.

In the mandible, the differences were the most pronounced, with an F-value of 11.810 and *p* = 0.000, indicating significant variations between groups and reduced variability within groups, reflecting clearer differences among examiner types. These results highlight that the sensitivity and experience of each group in image interpretation significantly influence the diagnosis, particularly in more complex cases such as periodontal space widening in the mandible. A post-hoc analysis could more clearly identify the groups responsible for these variations.

The Bonferroni analysis highlighted significant differences between groups in identifying periodontal space widening. At the general level, dentists differed significantly from senior specialists (mean difference = −0.35000, *p* = 0.011), indicating differing diagnostic approaches. In the maxilla, senior specialists showed significant differences compared to specialists and dentists (mean difference = 0.35000, *p* = 0.045), suggesting variations in maxillary imaging interpretation among these groups. The other comparisons, including those between AI and physicians, did not reveal significant differences at this level, as can be seen in Table 8.

In the mandible, dentists showed the largest differences, compared to both senior specialists (mean difference = −0.50000, *p* = 0.000) and specialists (mean difference = −0.40000, *p* = 0.004), highlighting greater variability in identifying mandibular pathologies. In contrast, AI did not demonstrate significant differences compared to any group, indicating relative consistency in assessments and suggesting that AI’s sensitivity may be similar to that of other examiners. These results emphasize the need for standardization in the interpretation of orthopantomograms, particularly for more complex cases such as those involving the mandible.

### 3.4. Assessing Bone Loss

The analysis of participant bone loss detection revealed notable differences in the ability and consistency of each group (Table 9). Senior specialists and artificial intelligence (AI) demonstrated perfect bone loss detection capabilities, with a mean of 1.00 and no variation (standard deviation, 0.00), indicating complete uniformity in accurately identifying cases. Specialists achieved a mean of 0.95, reflecting a slight reduction in accuracy, with a standard deviation of 0.22, signaling moderate variability in interpretations.

In contrast, dentists faced the greatest challenges in detecting bone loss, particularly in the maxilla, where their mean dropped to 0.70 with a significant standard deviation of 0.47, indicating high variability and some low detection rates. At the mandibular level, the dentists’ mean improved to 0.85, but variability remained high (standard deviation, 0.36).

The ANOVA analysis highlighted significant differences only in the detection of bone loss in the maxilla. The statistical value F = 4.328 and the significance level *p* = 0.008 indicate significant differences between groups in detecting bone loss in the maxilla. For generalized bone loss and bone loss in the mandible, the significance values were higher (*p* = 0.218 for general and *p* = 0.259 for the mandible), suggesting that the differences between groups were not statistically significant (Table 10).

The multiple comparisons analysis (Bonferroni) revealed that the participants generally performed similarly in detecting generalized and mandibular bone loss, with no significant differences between groups. However, in the maxilla, there was a significant difference between senior specialists and dentists, with senior specialists showing better performance (mean difference = 0.30, *p* = 0.011). This suggests that assessing bone loss in the maxilla is more challenging, and dentists face greater difficulties in this area compared to the other participants. The results highlight the potential need for additional training or decision-support tools to improve the evaluation of maxillary bone loss, particularly for dentists, as shown in Table 11.

## 4. Discussion

There has been a significant rise in studies investigating the potential of AI models in periodontics, suggesting that this field may soon undergo a radical transformation due to the numerous ways in which AI is already being applied [42,43,44]. Over the course of their lifetimes, over 3.5 billion individuals will experience some type of oral illness, according to the World Health Organization [45]. Because of this, there is an immediate need for resources to help practitioners and expand people’s access to oral healthcare.

Many artificial intelligence decision support systems have been trained on panoramic radiographs, and this type of imaging is now standard practice in dentistry pre-treatment screenings [46].

The findings of this study highlight the potential and limitations of artificial intelligence (AI) in periodontal assessment compared to human expertise. By examining the performance of AI alongside senior specialists, specialists, and general dentists, this study revealed key insights into diagnostic accuracy, variability, and areas requiring improvement.

The use of orthopantomograms (OPGs) in this study was intentional despite the well-recognized gold standard of periapical radiographs for detailed periodontal diagnostics. OPGs were chosen due to their ability to provide a comprehensive view of the entire dentition and surrounding structures in a single image. This broad perspective is particularly advantageous in studies aiming to evaluate diagnostic consistency across practitioners and AI systems in complex periodontal conditions, such as generalized bone loss or furcation involvement. The limitations of OPGs in providing detailed clinical data are acknowledged, and the findings emphasize the need to complement radiographic evaluations with clinical examinations to ensure comprehensive periodontal diagnosis [47].

It is important to acknowledge that radiographic images alone cannot provide a complete periodontal assessment, as clinical parameters such as bleeding on probing, attachment loss, and gingival condition are not captured.

AI demonstrated a performance comparable to that of senior specialists, achieving high levels of consistency and accuracy in diagnosing complex periodontal conditions. This highlights the potential of AI to complement human expertise, particularly in identifying subtle indicators of periodontal disease that may be overlooked in manual evaluations. Senior specialists, owing to their extensive experience, consistently outperformed the other groups, reinforcing the importance of clinical expertise in diagnostic accuracy.

It can be difficult, time-consuming, and subjective for the examiner to calculate radiographic bone loss. Algorithms based on artificial intelligence have been developed to automatically detect radiographic bone loss and the probability of periodontal disease and tooth loss [48]. In their assessment of artificial intelligence models for periodontal disease detection, Miller et al. [49] looked at their ability to identify radiographic bone loss. Panoramic radiographs demonstrated an average accuracy ranging from 63% to 94%, while periapical radiographs showed 25% accuracy for diagnosing moderate illness and 99% accuracy for staging radiographic bone loss.

Our results indicate that AI performs well in detecting horizontal bone loss, with accuracy comparable to senior specialists and no statistically significant differences (*p* > 0.05). This suggests that AI is a reliable tool for assessing generalized alveolar bone resorption. These findings align with previous research, which has reported AI’s ability to standardize radiographic assessments and improve diagnostic reliability [50,51].

However, AI demonstrated greater variability in detecting vertical bone loss, particularly in the maxilla. While its performance was comparable to senior specialists and specialists, it showed more variation when compared to general dentists (*p* = 0.259). This suggests that AI may have limitations in identifying complex vertical defects, such as deep angular bone loss or furcation involvement, which require further refinement in its algorithms.

Several studies have evaluated AI’s role in dental diagnostics. A systematic review by Revilla-León et al. [52] found that AI models achieved high accuracy in identifying alveolar bone loss from radiographs, consistent with our findings. Similarly, Scott et al. [53] reported that AI-based periodontal assessments reduce human error and improve early disease detection, supporting the notion that AI can act as a decision-support tool in clinical settings.

In conclusion, AI is highly effective for detecting horizontal bone loss, aligning well with expert assessments, but its reliability in vertical bone loss detection remains inconsistent. Future improvements in AI models could enhance their ability to detect complex bone defects with higher accuracy.

This study found significant differences in diagnostic performance depending on the anatomical location. Maxillary bone loss posed greater challenges for general dentists, as reflected in lower mean scores and higher variability. Conversely, mandibular assessments showed less pronounced differences among groups, though AI and senior specialists still demonstrated superior performance. These findings suggest that anatomical complexity and radiographic interpretation may influence diagnostic variability.

While our study establishes that AI demonstrates statistically significant superiority in periodontal assessment compared to less experienced practitioners, its clinical significance must be interpreted in the context of patient-centered care. AI has the potential to enhance diagnostic precision, reduce variability, and support clinical decision-making, but its impact on real-world patient outcomes requires further investigation.

AI should complement human expertise, not replace it, as clinical decisions involve factors beyond imaging analysis. Future studies should focus on longitudinal assessments and real-world applications to validate AI’s effectiveness in improving periodontal disease assessment.

Algorithmic bias and limited generalizability across diverse populations have been widely discussed in recent literature [54,55]. These biases often stem from the datasets used for training AI models, which may not adequately represent the diversity of patient populations. For instance, disparities in radiographic imaging quality, variations in anatomical structures across ethnic groups, or underrepresentation of certain clinical conditions can skew AI performance [56,57]. These biases could lead to misdiagnosis or underdiagnosis in specific patient subsets, potentially exacerbating healthcare inequalities. Addressing this issue requires continuous validation of AI algorithms using diverse, high-quality datasets that reflect the heterogeneity of real-world populations [58].

Another significant challenge lies in the generalizability of AI models across diverse clinical settings. AI systems trained on specific datasets may struggle to adapt when exposed to variations in imaging techniques, equipment, or clinical protocols [59]. This limitation highlights the necessity for rigorous testing and standardization of AI tools before their widespread adoption.

Moreover, efforts to minimize algorithmic bias and improve the adaptability of AI to diverse patient populations are essential for its broader acceptance and effectiveness. Combining AI diagnostics with real-time clinical data, such as patient histories and intraoral findings, could further enhance its precision and applicability [60].

Another limitation of this study is the selection of six high-quality panoramic radiographs (OPGs) for evaluation. While this ensured a controlled and standardized assessment, it may have led to an overestimation of AI’s diagnostic accuracy. In clinical practice, radiographic quality can vary due to factors such as patient positioning, exposure settings, and imaging artifacts. Future studies should include a broader range of image quality to better evaluate AI performance under real-world conditions and ensure its applicability across diverse clinical scenarios.

In addition to these broader considerations, this study has several limitations that should be acknowledged. First, the use of a limited sample size and a specific AI platform (Planmeca Romexis) may constrain the generalizability of the findings. Future studies should expand the sample size and include multiple AI systems to provide a more comprehensive evaluation. Second, this study focused on static radiographic images, which may not capture the full complexity of periodontal conditions. Incorporating dynamic imaging modalities or integrating clinical examination data could offer a more holistic assessment of AI’s diagnostic capabilities.

Another limitation is the absence of longitudinal data to evaluate AI’s performance over time. Periodontal disease progression is a dynamic process, and longitudinal studies would provide valuable insights into AI’s ability to monitor changes and guide treatment planning effectively. Furthermore, while the statistical analyses used in this study were robust, additional methods, such as machine learning interpretability techniques, could enhance the understanding of how AI arrives at specific diagnoses.

Lastly, this study did not explore the ethical and regulatory challenges associated with AI adoption in dentistry. Issues such as data privacy, accountability for AI-driven decisions, and the potential for over-reliance on technology warrant further investigation. Establishing clear guidelines and frameworks for the ethical use of AI in clinical settings is crucial to ensuring patient trust and safety.

## 5. Conclusions

This study provided valuable insights into the comparative performance of artificial intelligence (AI) and human practitioners in periodontal diagnostics, focusing on key clinical parameters such as attachment loss, periodontal pockets, and alveolar bone loss. The findings have implications for clinical practice, education, and the future integration of AI technologies in dentistry.

The results emphasize the importance of integrating AI into clinical workflows to enhance diagnostic accuracy and efficiency. For general dentists and specialists, AI can act as a supplementary tool to bridge gaps in expertise. Additionally, training programs focusing on advanced radiographic interpretation and the use of AI tools could further improve diagnostic outcomes.

Further studies should explore the integration of AI with real-time clinical data to assess its impact on treatment outcomes. Longitudinal studies examining the role of AI in reducing diagnostic errors and improving patient care are also warranted. By integrating AI into clinical workflows and addressing its current limitations, dentistry can move toward a future where technology and human expertise work hand-in-hand to improve patient outcomes.

While our findings contribute to the understanding of AI’s role in periodontal diagnostics, further studies should explore its performance across diverse clinical scenarios. The continuous refinement of AI models, expansion of datasets to include real-world imaging variability, and integration of clinical parameters will enhance the reliability of AI-assisted diagnostics. Future research should also address algorithmic biases and generalizability to different patient populations to ensure equitable and consistent outcomes. Acknowledging these challenges will allow AI to develop as a valuable tool that complements human expertise while improving diagnostic precision in clinical practice.

This study demonstrates that while AI holds significant promise in augmenting periodontal diagnostics, it should be viewed as a complementary tool rather than a replacement for human expertise.

## Figures and Tables

**Table 1:** The ANOVA test applied to determine the statistical differences between groups regarding the number of identified losses of attachment.

		Sum of Squares	df	Mean Square	F	Sig.
Teeth with attachment loss	Between groups	44.788	3	14.929	3.283	0.027
	Within groups	281.929	62	4.547		
	Total	326.717	65			
Loss of attachment of the maxillary arch	Between groups	40.176	3	13.392	3.820	0.014
	Within groups	7.369	62	3.506		
	Total	257.546	65			
Loss of attachment of the mandibular arch	Between groups	25.282	3	8.427	1.802	0.156
	Within groups	289.891	62	4.676		
	Total	315.174	65			

**Table 2:** The Bonferroni comparative analysis regarding the number of teeth with attachment loss between groups.

Dependent Variable	(I) Participant Type	(J) Participant Type	Mean Difference (I-J)	Std. Error	Sig.	95% Confidence Interval	
						Lower Bound	Upper Bound
Teeth with attachment loss	Senior specialists	Senior specialists	0.84400	0.67433	1.000	−0.9939	2.6819
		General dentists	1.77220	0.67433	0.065	−0.0657	3.6101
		AI	−0.69283	0.99259	1.000	−3.3982	2.0125
	Specialists	Senior specialists	−0.84400	0.67433	1.000	−2.6819	0.9939
		General dentists	0.92820	0.67433	1.000	−0.9097	2.7661
		AI	−1.53683	0.99259	0.760	−4.2422	1.1685
	General dentists	Senior specialists	−1.77220	0.67433	0.065	−3.6101	0.0657
		Specialists	−0.92820	0.67433	1.000	−2.7661	0.9097
		AI	−2.46503	0.99259	0.094	−5.1704	0.2403
	AI	Senior specialists	0.69283	0.99259	1.000	−2.0125	3.3982
		Specialists	1.53683	0.99259	0.760	−1.1685	4.2422
		General dentists	2.46503	0.99259	0.094	−0.2403	5.1704
Loss of attachment of the maxillary arch	Senior specialists	Medical specialist dentists	1.19900	0.59211	0.283	−0.4148	2.8128
		General dentists	1.62800 *	0.59211	0.047	0.0142	3.2418
		AI	−0.52200	0.87156	1.000	−2.8975	1.8535
	Specialists	Senior specialists	−1.19900	0.59211	0	−2.8128	0.4148
		General dentists	0.42900	0.59211	1.000	−1.1848	2.0428
		AI	−1.72100	0.87156	0.317	−4.0965	0.6545
	General dentists	Senior specialists	−1.62800 *	0.59211	0.047	−3.2418	−0.0142
		Specialists	−0.42900	0.59211	1.000	−2.0428	1.1848
		AI	−2.15000	0.87156	0.098	−4.5255	0.2255
	AI	Senior specialists	0.52200	0.87156	1.000	−1.8535	2.8975
		Specialists	1.72100	0.87156	0.317	−0.6545	4.0965
		General dentists	2.15000	0.87156	0.098	−0.2255	4.5255
Loss of attachment of the mandibular arch	Senior specialists	Specialists	0.58100	0.68379	1.000	−1.2827	2.4447
		General dentists	1.46900	0.68379	0.214	−0.3947	3.3327
		AI	−0.10483	1.00651	1.000	−2.8481	2.6385
	Specialists	Senior specialists	−0.58100	0.68379	1.000	−2.4447	1.2827
		General dentists	0.88800	0.68379	1.000	−0.9757	2.7517
		AI	−0.68583	1.00651	1.000	−3.4291	2.0575
	Dentists	Senior specialists	−1.46900	0.68379	0.214	−3.3327	0.3947
		Specialists	−0.88800	0.68379	1.000	−2.7517	0.9757
		AI	−1.57383	1.00651	0.738	−4.3171	1.1695
	AI	Senior specialists	0.10483	1.00651	1.000	−2.6385	2.8481
		Specialists	0.68583	1.00651	1.000	−2.0575	3.4291
		General dentists	1.57383	1.00651	0.738	−1.1695	4.3171

* The mean difference is significant at the 0.05 level.

**Table 3:** Descriptive analysis of the mean values of periodontal pockets per group.

		N	Mean	Std. Deviation	Std. Error	95% Confidence Interval for Mean		Minimum	Maximum
						Lower Bound	Upper Bound		
Teeth with periodontal pockets	Senior specialists	20	1.0000	0.00000	0.00000	1.0000	1.0000	1.00	1.00
	Specialists	20	0.9000	0.30779	0.06882	0.7559	1.0441	0.00	1.00
	General dentists	20	0.7500	0.44426	0.09934	0.5421	0.9579	0.00	1.00
	AI	6	0.8333	0.40825	0.16667	0.4049	1.2618	0.00	1.00
	Total	66	0.8788	0.32887	0.04048	0.7979	0.9596	0.00	1.00
Vertical pockets	Senior specialists	20	1.0000	0.00000	0.00000	1.0000	1.0000	1.00	1.00
	Specialists	20	0.7000	0.47016	0.10513	0.4800	0.9200	0.00	1.00
	General dentists	20	0.5000	0.51299	0.11471	0.2599	0.7401	0.00	1.00
	AI	6	0.8333	0.40825	0.16667	0.4049	1.2618	0.00	1.00
	Total	66	0.7424	0.44065	0.05424	0.6341	0.8507	0.00	1.00
Angled pockets	Senior specialists	20	1.0000	0.00000	0.00000	1.0000	1.0000	1.00	1.00
	Specialists	20	0.7000	0.57124	0.12773	0.4327	0.9673	0.00	2.00
	General dentists	20	0.3500	0.48936	0.10942	0.1210	0.5790	0.00	1.00
	AI	6	0.6667	0.51640	0.21082	0.1247	1.2086	0.00	1.00
	Total	66	0.6818	0.50105	0.06167	0.5586	0.8050	0.00	2.00

**Table 4:** The ANOVA test applied to determine the statistical differences between groups regarding the number of identified periodontal pockets.

		Sum of Squares	df	Mean Square	F	Sig.
Teeth with periodontal pockets	Between groups	20	1.0000	1.0000	1.00	1.00
	Within groups	20	0.9000	1.0441	0.00	1.00
	Total	20	0.7500	0.9579	0.00	1.00
Vertical pockets	Between groups	20	1.0000	1.0000	1.00	1.00
	Within groups	20	0.7000	0.9200	0.00	1.00
	Total	66	0.7424	0.8507	0.00	1.00
Angled pockets	Between groups	20	1.0000	1.0000	1.00	1.00
	Within groups	20	0.7000	0.9673	0.00	2.00
	Total	66	0.6818	0.8050	0.00	2.00

**Table 5:** The Bonferroni test for multiple comparisons was used to analyze the relationship between the number and type of detected periodontal pockets and participant groups.

Dependent Variable	(I) Participant Type	(J) Participant Type	Mean Difference (I-J)	Std. Error	Sig.	95% Confidence Interval	
						Lower Bound	Upper Bound
Teeth with periodontal pockets	Senior specialists	Specialists	0.10000	0.10147	1.000	−0.1766	0.3766
		General dentists	0.25000	0.10147	0.099	−0.0266	0.5266
		AI	0.16667	0.14936	1.000	−0.2404	0.5737
	Specialists	Senior specialists	−0.10000	0.10147	1.000	−0.3766	0.1766
		General dentists	0.15000	0.10147	0.866	−0.1266	0.4266
		AI	0.06667	0.14936	1.000	−0.3404	0.4737
	General dentists	Senior specialists	−0.25000	0.10147	0.099	−0.5266	0.0266
		Specialists	−0.15000	0.10147	0.866	−0.4266	0.1266
		AI	−0.08333	0.14936	1.000	−0.4904	0.3237
	AI	Senior specialists	−0.16667	0.14936	1.000	−0.5737	0.2404
		Specialists	−0.06667	0.14936	1.000	−0.4737	0.3404
		General dentists	0.08333	0.14936	1.000	−0.3237	0.4904
Vertical pockets	Senior specialists	Specialists	0.30000	0.12721	0.129	−0.0467	0.6467
		General dentists	0.50000 *	0.12721	0.001	0.1533	0.8467
		AI	0.16667	0.18725	1.000	−0.3437	0.6770
	Specialists	Senior specialists	−0.30000	0.12721	0.129	−0.6467	0.0467
		General dentists	0.20000	0.12721	0.726	−0.1467	0.5467
		AI	−0.13333	0.18725	1.000	−0.6437	0.3770
	General dentist	Senior specialists	−0.50000 *	0.12721	0.001	−0.8467	−0.1533
		Specialists	−0.20000	0.12721	0.726	−0.5467	0.1467
		AI	−0.33333	0.18725	0.480	−0.8437	0.1770
	AI	Senior specialists	−0.16667	0.18725	1.000	−0.6770	0.3437
		Specialists	0.13333	0.18725	1.000	−0.3770	0.6437
		General dentists	0.33333	0.18725	0.480	−0.1770	0.8437
Angled pockets	Senior specialists	Specialists	0.30000	0.13960	0.213	−0.0805	0.6805
		General dentists	0.65000 *	0.13960	0.000	0.2695	1.0305
		AI	0.33333	0.20549	0.659	−0.2267	0.8934
	Specialists	Senior specialists	−0.30000	0.13960	0.213	−0.6805	0.0805
		General dentists	0.35000	0.13960	0.089	−0.0305	0.7305
		AI	0.03333	0.20549	1.000	−0.5267	0.5934
	General dentists	Senior specialists	−0.65000 *	0.13960	0.000	−1.0305	−0.2695
		Specialists	−0.35000	0.13960	0.089	−0.7305	0.0305
		AI	−0.31667	0.20549	0.770	−0.8767	0.2434
	AI	Senior specialists	−0.33333	0.20549	0.659	−0.8934	0.2267
		Specialists	−0.03333	0.20549	1.000	−0.5934	0.5267
		General dentists	0.31667	0.20549	0.770	−0.2434	0.8767

* The mean difference is significant at the 0.05 level.

**Table 6:** Descriptive analysis of the mean values of teeth presenting periodontal space widening across groups.

		N	Mean	Std. Deviation	Std. Error	95% Confidence Interval for Mean		Minimum	Maximum
						Lower Bound	Upper Bound		
Widened PDL	Senior specialists	20	1.0000	0.00000	.00000	1.0000	1.0000	1.00	1.00
	Specialists	20	0.9000	0.30779	0.06882	0.7559	1.0441	0.00	1.00
	General dentists	20	0.6500	0.48936	0.10942	0.4210	0.8790	0.00	1.00
	AI	6	0.8333	0.40825	0.16667	0.4049	1.2618	0.00	1.00
	Total	66	0.8485	0.36130	0.04447	0.7597	0.9373	0.00	1.00
Widened maxillary PDL	Senior specialists	20	1.0000	0.00000	0.00000	1.0000	1.0000	1.00	1.00
	Specialists	20	0.6500	0.48936	0.10942	0.4210	0.8790	0.00	1.00
	General dentists	20	0.6500	0.48936	0.10942	0.4210	0.8790	0.00	1.00
	AI	6	0.8333	0.40825	0.16667	0.4049	1.2618	0.00	1.00
	Total	66	0.7727	0.42228	0.05198	0.6689	0.8765	0.00	1.00
Widened mandibular PDL	Senior specialists	20	1.0000	0.00000	0.00000	1.0000	1.0000	1.00	1.00
	Specialists	20	0.9000	0.30779	0.06882	0.7559	1.0441	0.00	1.00
	General dentists	20	0.5000	0.51299	0.11471	0.2599	0.7401	0.00	1.00
	AI	6	0.8333	0.40825	0.16667	0.4049	1.2618	0.00	1.00
	Total	66	0.8030	0.40076	0.04933	0.7045	0.9015	0.00	1.00

**Table 7:** The ANOVA test applied to determine the statistical differences between groups regarding periodontal space widening.

		Sum of Squares	df	Mean Square	F	Sig.
Widened PDL	Between Groups	1.302	3	0.434	3.744	0.015
	Within Groups	7.183	62	0.116		
	Total	8.485	65			
Widened maxillary PDL	Between Groups	1.658	3	0.553	3.449	0.022
	Within Groups	9.933	62	0.160		
	Total	11.591	65			
Widened mandibular PDL	Between Groups	2.806	3	0.935	7.597	0.000
	Within Groups	7.633	62	0.123		
	Total	10.439	65			

**Table 8:** The Bonferroni test for multiple comparisons was used to analyze the relationship between detected periodontal space widening and participant groups.

Dependent Variable	(I) Participant Type	(J) Participant Type	Mean Difference (I–J)	Std. Error	Sig.	95% Confidence Interval	
						Lower Bound	Upper Bound
Widened PDL	Senior specialists	Specialists	0.10000	0.10764	1.000	−0.1934	0.3934
		General dentists	0.35000 *	0.10764	0.011	0.0566	0.6434
		AI	0.16667	0.15844	1.000	−0.2652	0.5985
	Specialists	Senior specialists	−0.10000	0.10764	1.000	−0.3934	0.1934
		General dentists	0.25000	0.10764	0.141	−0.0434	0.5434
		AI	0.06667	0.15844	1.000	−0.3652	0.4985
	General dentists	Senior specialists	−0.35000 *	0.10764	0.011	−0.6434	−0.0566
		Specialists	−0.25000	0.10764	0.141	−0.5434	0.0434
		AI	−0.18333	0.15844	1.000	−0.6152	0.2485
	AI	Senior specialists	−0.16667	0.15844	1.000	−0.5985	0.2652
		Specialists	−0.06667	0.15844	1.000	−0.4985	0.3652
		General dentists	0.18333	0.15844	1.000	−0.2485	0.6152
Widened maxillary PDL	Senior specialists	Specialists	0.35000 *	0.12658	0.045	0.0050	0.6950
		Dentists	0.35000 *	0.12658	0.045	0.0050	0.6950
		AI	0.16667	0.18631	1.000	−0.3411	0.6745
	Specialists	Senior specialists	−0.35000 *	0.12658	0.045	−0.6950	−0.0050
		General dentists	0.00000	0.12658	1.000	−0.3450	0.3450
		AI	−0.18333	0.18631	1.000	−0.6911	0.3245
	General dentists	Senior specialists	−0.35000 *	0.12658	0.045	−0.6950	−0.0050
		Specialists	0.00000	0.12658	1.000	−0.3450	0.3450
		AI	−0.18333	0.18631	1.000	−0.6911	0.3245
	AI	Senior specialists	−0.16667	0.18631	1.000	−0.6745	0.3411
		Specialists	0.18333	0.18631	1.000	−0.3245	0.6911
		General dentists	0.18333	0.18631	1.000	−0.3245	0.6911
Widened mandibular PDL	Senior specialists	Specialists	0.10000	0.11096	1.000	−0.2024	0.4024
		General dentists	0.50000 *	0.11096	0.000	0.1976	0.8024
		AI	0.16667	0.16333	1.000	−0.2785	0.6118
	Specialists	Senior specialists	−0.10000	0.11096	1.000	−0.4024	0.2024
		General dentists	0.40000 *	0.11096	0.004	0.0976	0.7024
		AI	0.06667	0.16333	1.000	−0.3785	0.5118
	General dentists	Senior specialists	−0.50000 *	0.11096	0.000	−0.8024	−0.1976
		Specialists	−0.40000 *	0.11096	0.004	−0.7024	−0.0976
		AI	−0.33333	0.16333	0.273	−0.7785	0.1118
	AI	Senior specialists	−0.16667	0.16333	1.000	−0.6118	0.2785
		Specialists	−0.06667	0.16333	1.000	−0.5118	0.3785
		General dentists	0.33333	0.16333	0.273	−0.1118	0.7785

* The mean difference is significant at the 0.05 level.

**Table 9:** Descriptive analysis of detected bone loss across participant groups.

		N	Mean	Std. Deviation	Std. Error	95% Confidence Interval for Mean		Minimum	Maximum
						Lower Bound	Upper Bound		
Alveolar bone loss	Senior specialists	20	1.0000	0.00000	0.00000	1.0000	1.0000	1.00	1.00
	Specialists	20	0.9500	0.22361	0.05000	0.8453	1.0547	0.00	1.00
	General dentists	20	0.8500	0.36635	0.08192	0.6785	1.0215	0.00	1.00
	AI	6	1.0000	0.00000	0.00000	1.0000	1.0000	1.00	1.00
	Total	66	0.9394	0.24043	0.02960	0.8803	0.9985	0.00	1.00
Alveolar bone loss in the maxilla	Senior specialists	20	1.0000	0.00000	0.00000	1.0000	1.0000	1.00	1.00
	Specialists	20	0.9500	0.22361	0.05000	0.8453	1.0547	0.00	1.00
	General dentists	20	0.7000	0.47016	0.10513	0.4800	0.9200	0.00	1.00
	AI	5	1.0000	0.00000	0.00000	1.0000	1.0000	1.00	1.00
	Total	65	.8923	0.31240	0.03875	0.8149	0.9697	0.00	1.00
Alveolar bone loss in the mandible	Senior specialists	20	1.0000	0.00000	0.00000	1.0000	1.0000	1.00	1.00
	Specialists	20	0.9500	0.22361	0.05000	0.8453	1.0547	0.00	1.00
	General dentists	20	0.8500	0.36635	0.08192	0.6785	1.0215	0.00	1.00
	AI	6	0.8333	0.40825	0.16667	0.4049	1.2618	0.00	1.00
	Total	66	0.9242	0.26664	0.03282	0.8587	0.9898	0.00	1.00

**Table 10:** The ANOVA test applied to determine the statistical difference between groups regarding alveolar bone loss.

		Sum of Squares	df	Mean Square	F	Sig.
Alveolar bone loss	Between groups	0.258	3	0.086	1.521	0.218
	Within groups	3.500	62	0.056		
	Total	3.758	65			
Alveolar bone loss in the maxilla	Between groups	1.096	3	0.365	4.328	0.008
	Within groups	5.150	61	0.084		
	Total	6.246	64			
Alveolar bone loss in the mandible	Between groups	0.288	3	0.096	1.373	0.259
	Within groups	4.333	62	0.070		
	Total	4.621	65			

**Table 11:** Bonferroni comparative analysis regarding the number of teeth with bone loss across groups.

Dependent Variable	(I) Participant Type	(J) Participant Type	Mean Difference (I–J)	Std. Error	Sig.	95% Confidence Interval	
						Lower Bound	Upper Bound
Alveolar bone loss	Senior specialists	Specialists	0.05000	0.07513	1.000	−0.1548	0.2548
		General dentists	0.15000	0.07513	0.302	−0.0548	0.3548
		AI	0.00000	0.11059	1.000	−0.3014	0.3014
	Specialists	Senior specialists	−0.05000	0.07513	1.000	−0.2548	0.1548
		General dentists	0.10000	0.07513	1.000	−0.1048	0.3048
		AI	−0.05000	0.11059	1.000	−0.3514	0.2514
	General dentists	Senior specialists	−0.15000	0.07513	0.302	−0.3548	0.0548
		Specialists	−0.10000	0.07513	1.000	−0.3048	0.1048
		AI	−0.15000	0.11059	1.000	−0.4514	0.1514
	AI	Senior specialists	0.00000	0.11059	1.000	−0.3014	0.3014
		Specialists	0.05000	0.11059	1.000	−0.2514	0.3514
		General dentists	0.15000	0.11059	1.000	−0.1514	0.4514
Alveolar bone loss in the maxilla	Senior specialists	Specialists	0.05000	0.09188	1.000	−0.2006	0.3006
		General dentists	0.30000 *	0.09188	0.011	0.0494	0.5506
		AI	0.00000	0.14528	1.000	−0.3962	0.3962
	Specialists	Senior specialists	−0.05000	0.09188	1.000	−0.3006	0.2006
		General dentists	0.25000	0.09188	0.051	−0.0006	0.5006
		AI	−0.05000	0.14528	1.000	−0.4462	0.3462
	General dentists	Senior specialists	−0.30000 *	0.09188	0.011	−0.5506	−0.0494
		Specialists	−0.25000	0.09188	0.051	−0.5006	0.0006
		AI	−0.30000	0.14528	0.259	−0.6962	0.0962
	AI	Senior specialists	0.00000	0.14528	1.000	−0.3962	0.3962
		Specialists	0.05000	0.14528	1.000	−0.3462	0.4462
		General dentists	0.30000	0.14528	0.259	−0.0962	0.6962
Alveolar bone loss in the mandible	Senior specialists	Specialists	0.05000	0.08360	1.000	−0.1779	0.2779
		General dentists	0.15000	0.08360	0.466	−0.0779	0.3779
		AI	0.16667	0.12306	1.000	−0.1687	0.5021
	Specialists	Senior specialists	−0.05000	0.08360	1.000	−0.2779	0.1779
		General dentists	0.10000	0.08360	1.000	−0.1279	0.3279
		AI	0.11667	0.12306	1.000	−0.2187	0.4521
	General dentists	Senior specialists	−0.15000	0.08360	0.466	−0.3779	0.0779
		Specialists	−0.10000	0.08360	1.000	−0.3279	0.1279
		AI	0.01667	0.12306	1.000	−0.3187	0.3521
	AI	Senior specialists	−0.16667	0.12306	1.000	−0.5021	0.1687
		Specialists	−0.11667	0.12306	1.000	−0.4521	0.2187
		General dentists	−0.01667	0.12306	1.000	−0.3521	0.3187

* The mean difference is significant at the 0.05 level.

## Data Availability

All data are available from the corresponding author upon reasonable request.
